# Detection of Mimivirus from respiratory samples in tuberculosis-suspected patients

**DOI:** 10.1038/s41598-022-12757-6

**Published:** 2022-05-23

**Authors:** Fatemeh Sakhaee, Jalal Mosayebi Amroabadi, Sara Razi, Farzam Vaziri, Farid Abdolrahimi, Sina Moghaddam, Fatemeh Rahimi Jamnani, Seyed Davar Siadat, Abolfazl Fateh

**Affiliations:** 1 Department of Mycobacteriology and Pulmonary Research, Pasteur Institute of Iran, Tehran, Iran; 2 Artificial Intelligence and Multi-Omics Center (AIMOC), Stavanger, Norway; 3 Vira Pioneers of Modern Science (VIPOMC), Tehran, Iran; 4 Microbiology Research Center (MRC), Pasteur Institute of Iran, Tehran, Iran

**Keywords:** Diseases, Medical research, Pathogenesis

## Abstract

Acanthamoeba polyphaga mimivirus (APMV), a species of amoeba-infecting giant viruses, has recently emerged as human respiratory pathogens. This study aimed to evaluate the presence of Mimivirus in respiratory samples, collected from tuberculosis (TB)-suspected patients. The study was performed on 10,166 clinical respiratory samples from April 2013 to December 2017. Mimivirus was detected using a suicide nested-polymerase chain reaction (PCR) and real-time PCR methods. Of 10,166 TB-suspected patients, 4 (0.04%) were positive for Mimivirus*,* including Mimivirus-53, Mimivirus-186, Mimivirus-1291, and Mimivirus-1922. Three out of four patients, hospitalized in the intensive care unit (ICU), were mechanically ventilated. All patients had an underlying disease, and the virus was detected in both sputum and bronchoalveolar lavage samples. In conclusion, Mimivirus was isolated from TB-suspected patients in a comprehensive study. The present results, similar to previous reports, showed that Mimiviruses could be related to pneumonia. Further studies in different parts of the world are needed to additional investigate the clinical importance of Mimivirus infection.

## Introduction

The field of virology was transformed in 2003 with the accidental discovery of giant viruses of amoeba. These viruses were antecedently described in terms of their submicroscopic size, which can be determined by optical microscopy. Several reports have shown that giant viruses of amoebae have structural, genetic, and proteomic complexities, which are comparable to those of bacteria and some small eukaryotes and are not expected among viruses. Up to now, Mimivirus*,* mollivirus*,* faustovirus*,* marseillevirus, and pithovirus, as giant viruses of amoebae, have been increasingly identified in humans^1^.

Acanthamoeba polyphaga mimivirus (APMV) was introduced in 2005 as the main member of the family *Mimiviridae*. Since then, approximately 100 new Mimivirus strains have been detected by culturing amoebae from soil, water, and human samples^2^.

These viruses are intra-amoebal pathogens, which are isolated by culturing amoeba from water samples, collected from cooling towers^3,4^. They may also be causative agents of pneumonia, similar to other amoeba-associated microorganisms (AAMs). Overall, identification of etiological agents is very important in both community- and hospital-acquired pneumonia, because these infections are the leading cause of morbidity and mortality worldwide^5^.

In previous studies, to determine whether Mimivirus is a causative agent of pneumonia or not, it was inoculated via intranasal routes in mice, and histopathological evidence of acute pneumonia was found in most animals. The results of these studies suggest that this virus can cause pneumonia under experimental conditions^6^.

However, there is scarce information about the prevalence of Mimiviruses worldwide to determine the mechanisms of diseases caused by these viruses. Also, the frequency of Mimivirus has not been detected in Iranian patients.

Considering the possible role of Mimivirus as an etiological agent of pathogenic respiratory disease, this study aimed to detect Mimivirus DNA in tuberculosis (TB)-suspected patients, referred to the Department of Mycobacteriology and Pulmonary Research of Pasteur Institute of Iran.

## Materials and methods

### Study population

From April 2013 to December 2017, a total of 10,166 TB-suspected patients, presenting to Pasteur Institute of Iran, were selected. The current study was performed according to the 1975 Declaration of Helsinki and local regulations. It was also approved by the Ethics Committee of Pasteur Institute of Iran. Written informed consent was obtained from all participants and parents/legal guardians.

### Detection of Mimivirus by nested-polymerase chain reaction (PCR) and real-time PCR assays

Viral DNA and RNA were extracted from 10,166 respiratory tract samples, including sputum and bronchoalveolar lavage (BAL) samples, using the High Pure Viral Nucleic Acid Kit and the High Pure Viral RNA Kit (Roche Diagnostics Deutschland GmbH, Mannheim, Germany), according to the manufacturers’ instructions, respectively. Also, bacterial and fungal genomes were isolated using Proba-NK DNA Extraction Kit (DNA-Technology Company, Moscow, Russia) and the QIAamp^®^ DNA Mini Kit (QIAGEN, Carlsbad, USA), following the manufacturer’s instructions, respectively.

Also, a suicide nested-PCR assay was used for the identification of Mimivirus DNA in respiratory samples, as previously described^5^.

For the real-time PCR assay, two different conserved regions of the Mimivirus genome were targeted, according to a studies by Dare et al., and Saadi et al., as previously described^7,8^. Because we did not have access to Mimivirus as a positive control, we prepared a plasmid, containing amplicons as the positive control; also, distilled water was used as the negative control.

To confirm the results of PCR assays, an AccuPrep^®^ PCR Purification Kit (Bioneer, South Korea) was used to purify nested-PCR and real-time PCR products. Next, PCR products were sequenced using an ABI automated sequencer (Applied Biosystems, Foster City, CA, USA). MEGA Version 6.0 was used to evaluate the raw sequencing data.

### Phylogenetic analysis of Mimivirus based on core genes

For evaluating the evolutionary relationship between our isolated viruses and other families of Mimiviruses, phylogenetic analyses were performed, using nucleocytoplasmic large DNA virus (NCLDV) core genes, including major capsid protein, D5 helicase, family B-DNA polymerase, and VV A18 helicase^9^.

The PCR method was used for sequencing these genomes, using several overlapping amplicons. Two, five, three, and two fragments covering the near full-length genes of the VV A18 helicase, the family B-DNA polymerase, the D5 helicase, and the major capsid protein, respectively, were amplified by PCR using specific primers from Megavirus LBA111 (JX885207.1) isolate (Supplementary Tables 1–8). After purification with a High Pure PCR Product Purification Kit (Roche Diagnostics Deutschland GmbH, Mannheim, Germany), the purified PCR fragments were sequenced, using an ABI automated sequencer (Applied Biosystems, Foster City, CA, USA). MEGA Version 6.0 was used to evaluate the raw sequencing data.

## Results

**Figure 1:**
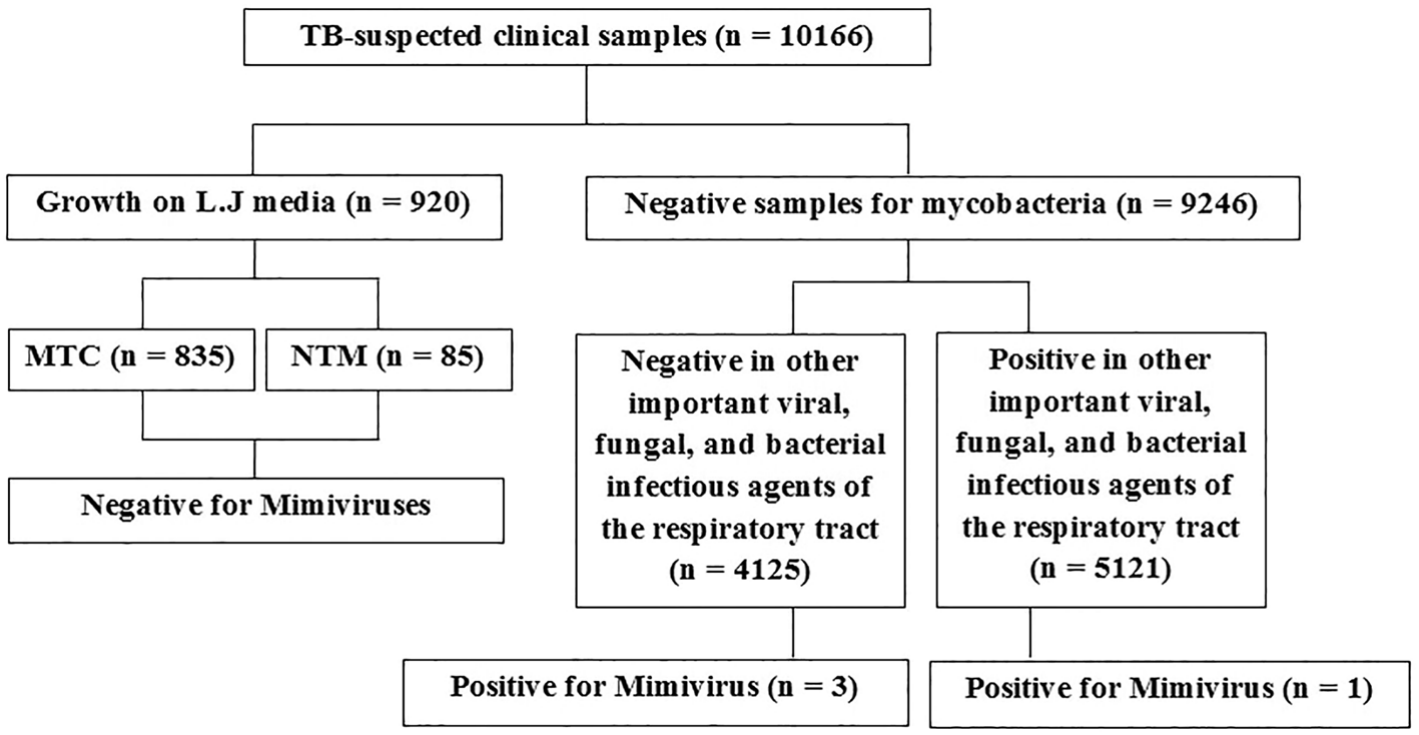
Flow chart of the study population selection. TB, Tuberculosis; MTC, *Mycobacterium tuberculosis* complex; NTM, nontuberculous mycobacteria.

**Figure 2:**
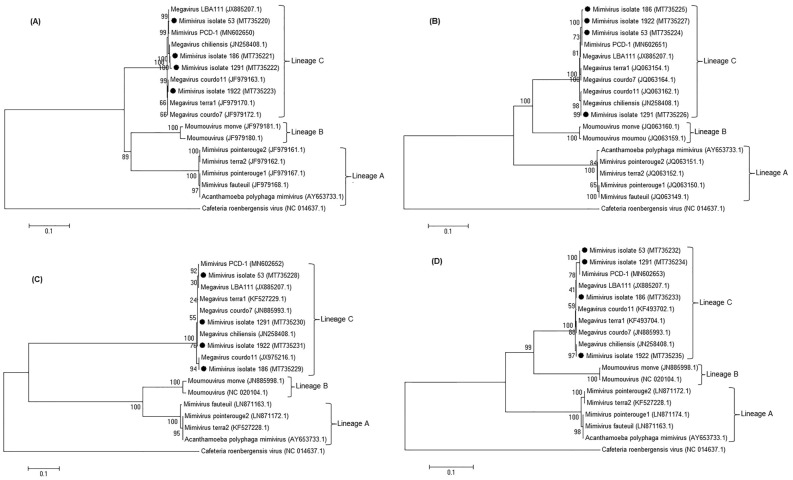
Neighbor-joining tree constructed based on nucleotide acid sequences of the family B DNA polymerase (**A**), the D5-ATPase-helicase (**B**), the major capsid protein (**C**), and the VV A18 helicase (**D**) genes. Outgroup was Cafeteria roenbergensis virus. Our isolated Mimiviruses are present within the Mimivirus C lineage.

Out of 10,166 TB-suspected patients, four (0.04%) were infected with Mimivirus (Fig. 1). In this study, the best results were obtained using the proposed method by Saadi and colleagues. Also, the result of suicide nested-PCR was positive for all positive samples and negative for the negative controls. The first- and second-round PCR products were 297 bp and 170 bp, respectively, visualized by electrophoresis on 1.5% agarose gel (Supplementary Fig. 1). After sequencing the PCR products that is shown in Supplementary Fig. 2A,B, the phylogenetic tree was drawn. The trees based on the core genes indicated the close relationship of all isolated Mimiviruses with Mimiviruses of lineage C and revealed that these isolates belonged to *Mimiviridae* clade C (Mimivirus chilensis, Megavirus LBA111, and Courdo11) (Fig. 2).

All positive samples were examined to determine the most common viral, fungal, and bacterial infectious agents of the respiratory tract, that is, *Mycobacterium tuberculosis*, nontuberculous mycobacteria, *Legionella pneumophila*, *Pseudomonas aeruginosa*, *Klebsiella pneumoniae*, *Streptococcus pneumoniae*, *Acinetobacter baumannii*, *Haemophilus influenzae*, *Staphylococcus aureus*, *Staphylococcus epidermidis*, *Proteus mirabilis*, *Escherichia coli*, *Serratia marcescens*, *Moraxella catarrhalis*, *Enterobacter aerogenes*, *Enterobacter cloacae*, *Citrobacter freundii*, *Citrobacter koseri*, *Stenotrophomonas maltophilia*, *Bacteroides fragilis*, *Prevotella intermedia*, *Candida albicans, Aspergillus* spp,* Mucor* spp, cytomegalovirus, herpes simplex virus type 1, influenza A and B viruses, respiratory syncytial virus, coronaviruses, rhinoviruses, parainfluenza viruses, and adenoviruses. In three positive samples, culture and PCR methods were negative for all bacterial, fungal, and viral agents (only PCR test performed for viral agents), except for one sample that had co-infection with *Legionella pneumophila* (Fig. 1).

The patient infected with Mimivirus-53 was a 25-year-old woman with renal disease, and her signs included fever, 10-kg weight loss, cough, night sweats, chest pain, and shortness of breath. The patient had been mechanically ventilated for more than eight days in the intensive care unit (ICU). Her clinical parameters showed an elevated white blood cell (WBC) count, erythrocyte sedimentation rate (ESR), and C-reactive protein (CRP) level. The level of vitamin D3 was below normal (Table 1). Computed tomography scan (CTS) indicated consolidation of the left lower lobe and bilateral basilar infiltrates. All pneumonia agents were negative for this patient. Out of three sputum samples, two samples were positive for Mimivirus, and the cycle threshold (Ct) value was 22 in the samples (Supplementary Fig. 3). Treatment with azithromycin was initiated for the patient, but the symptoms did not improve after 20 days, and she recovered slowly.

**Table 1:** Comparison laboratory parameters between all isolated Mimiviruses.

Variables	Mimivirus-53	Mimivirus-186	Mimivirus-1291	Mimivirus-1922
Age (years)	25	64	40	12
Gender	Female	Male	Male	Male
ALT, IU/L (Reference range: 5–40)	35	38	28	12
AST, IU/L (Reference range: 5–40)	33	36	24	15
Cholesterol, mg/dL (Reference range: 50–200)	156	195	210	120
TG, mg/dL (Reference range: 60–165)	145	145	165	95
LDL, mg/dL (Reference range: up to 150)	135	165	170	110
HDL, mg/dL (Reference range: > 40)	35	32	40	23
**WBC, 10**^9^**/L (Reference range: 4000–10,000)**	118,000	122,000	141,000	137,000
RBC, × 10^6^/µL (Reference range: 4.2–6.2)	4.5	5.2	5.3	4.8
**ESR, mm/1st h (Reference range: 0–15)**	34	42	98	78
FBS, mg/dL (Reference range: 70–100)	80	125	95	87
Urea, mg/dL (Reference range: 15–45)	55	31	25	17
Creatinine, mg/dL (Reference range: 0.6–1.4)	2.1	0.9	1.1	0.7
Uric acid, mg/dL (Reference range: 2.5–7.7)	8.2	3.2	4.1	2.8
Total bilirubin, mg/dL (Reference range: 0.2–1.2)	2.1	0.4	0.6	0.3
Direct bilirubin, mg/dL (Reference range: 0–0.2)	1.0	0.1	0.2	0.1
T3, ng/dL (Reference range: 2.3–4.2)	2.7	3.1	2.9	2.5
T4, μg/dL (Reference range: 5.6–13.7)	10.8	11.2	8.5	7.1
TSH, μ/L (Reference range: 0.4–4.5)	3.2	2.9	4.1	0.7
Ferritin, ng/mL (Reference range: 18–270)	345	175	125	145
Hemoglobin, g/dL (Reference range: 12–18)	11.5	14	17	13
Sodium, mEq/L (Reference range: 134–148)	147	137	140	135
Potassium, mEq/L (Reference range: 3.5–5.3)	4.2	3.8	4.1	3.6
Calcium, mg/dL (Reference range: 8.6–10.3)	8.8	9.2	9.7	8.9
Phosphorus, mg/dL (Reference range: 2.6–4.5)	5.1	2.9	3.4	3.2
**CRP, mg/L (Reference range: < 10 mg/L Negative)**	14.4	16.2	13.0	18.6
**25-hydroxyvitamin D, ng/mL (Sufficiency: 21–150)**	16.2	18	17.5	20.2
Underlying disease	Renal disease	Diabetes	HIV and HBV	Cystic fibrosis
Samples	Sputum (n = 3)	BAL (n = 3)	Sputum and BAL	Sputum and BAL

*ALT* alanine aminotransferase, *AST* aspartate aminotransferase, *ALP* alkaline phosphatase, *TG* triglyceride, *LDL* low density lipoprotein, *HDL* high density lipoprotein, *WBC* white blood cells, *RBC* red blood cells, *ESR* erythrocyte sedimentation rate, *FBS* fasting blood glucose, *T3* triiodothyronine, *T4* thyroxine, *TSH* thyroid-stimulating hormone, *LH* Luteinizing hormone, *FSH* follicle-stimulating hormone, *CRP* C-reactive protein, *HIV* human immunodeficiency virus, *HBV* hepatitis B virus. *The number of sputum and BAL samples was positive, respectively.

The patient with Mimivirus-186 was a diabetic 64-year-old man, presenting with fever, dyspnea, cough, and weight loss. He was diagnosed with pulmonary tuberculosis 5 years ago. The physician speculated the recurrence of *Mycobacterium tuberculosis* (M.tb) infection, but the results of smear, culture, and PCR tests for M.tb were negative. Also, all pneumonia agents were negative in the patient. He had an acute respiratory failure with the elevation of WBC count, CRP, and ESR (Table 1). CTS indicated peripheral subpleural opacity. After the evaluation of three BAL samples, all of them were found to be positive for Mimivirus. Also, the Ct values for BAL samples ranged from 18 to 20 (Supplementary Fig. 3).

The patient infected with Mimivirus-1291 was a 40-year-old man with human immunodeficiency virus (HIV), CD4 cell count below 300 cells/μL, and hepatitis B virus. Six months ago, he was admitted for treatment of *Pneumocystis carinii* pneumonia and oral candidiasis. During treatment, he had been mechanically ventilated for 12 days in the ICU. After 6 months of treatment, he complained of cough, fever, and night sweats with excessive sputum. Three sputum and two BAL samples were evaluated for pneumonia agents, but all important agents were negative. Two out of three sputum samples and two BAL samples were positive for Mimivirus infection. The Ct values were 19 and 21 for BAL and sputum samples, respectively (Supplementary Fig. 3). CTS indicated bilateral basilar infiltrates, suggesting viral pneumonia. Also, he showed increased WBC count, ESR, and CRP levels (Table 1).

The patient infected with Mimivirus-1922 was a 12-year-old boy with cystic fibrosis (CF). The CF genotype indicated a p.F508del mutation (ΔF508), and he was diagnosed with severe CF-related pulmonary disease. He was frequently admitted to the ICU and had been ventilated several times for a bacterial infection. Six months after discharge from the hospital, he showed signs of sputum production, weakness, night perspiration, cough, and fever. His biological parameters indicated elevated WBC count, ESR, and CRP levels (Table 1). CTS indicated multifocal consolidative changes and bilateral basilar infiltrates. The samples were negative for all pneumonia agents, except *Legionella pneumophila*. He was treated with azithromycin and levofloxacin, but his signs did not improve. All samples were evaluated for Mimivirus infection. Two BAL and two sputum samples with Ct values of 21 and 27 were positive for this virus, respectively, and the patient died unfortunately (Supplementary Fig. 3).

## Discussion

To the best of our knowledge, the current study was the first to investigate the presence of Mimivirus in TB-suspected patients with pulmonary signs.

In nearly 20–50% of patients, the causative agent of pneumonia, as the leading cause of infection-related mortality worldwide, is unknown. Therefore, a major public health goal is to distinguish new agents, causing both community- and hospital-acquired pneumonia^5^. Moreover, the correlation between human diseases and viral infections needs to be evaluated, as the cause of many pneumonia cases is not yet determined^10^.

Water-borne pathogens colonize water sources in hospitals, and some of them may be correlated with amoeba, such as APMV^10^. According to several reports, the presence of Mimivirus-specific antibodies is more common in nosocomial pneumonia patients, particularly in ICUs, compared to the controls^5,10^. Also, patients with community-acquired pneumonia and serological evidence of APMV are usually re-hospitalized after discharge, perhaps owing to insufficient administration of drugs against viral infections^10^.

According to several studies, even if controversial, there is some evidence that Mimiviruses may be a causative agent of pneumonia, especially in patients, who are hospitalized in ICUs and are mechanically ventilated for a long time^5,10,11^. To confirm this hypothesis, the results of the present study indicated that three out of four patients were hospitalized in ICUs and mechanically ventilated. Also, several serological reports indicated the higher percentage of seroconversion to *Mimiviruses* in ventilator-associated pneumonia patients, compared to patients without ventilator-associated pneumonia^11,12^.

In the current study, Mimivirus was accompanied by *Legionella pneumophila* in one patient with CF. Overall, contamination with microbial agents of water supplies has been associated with both hospital- and community-acquired pneumonia outbreaks^13^. Bacteria, such as *Burkholderia*, *Acinetobacter*, *Stenotrophomonas*, *Legionella*, and *Pseudomonas* species, have been detected in hospital water supplies. Some of these species, including *Legionella pneumophila*, have been correlated with hospital- and community-acquired pneumonia, and also have been related to free-living amoebas in hospital and natural aquatic environments^14–16^. A previous study reported that patients with nosocomial pneumonia, who received care next to a contaminated water distribution system, had antibodies against amoeba pathogens^17^. Also, seroconversion to *Acanthamoeba-*surviving bacteria was detected in 40% of patients, hospitalized in ICUs, in association with ventilator-associated pneumonia, particularly in the absence of identified infectious agents. Mimivirus*,* as a member of the group of intra-amoebal microorganisms, could be considered a potential agent of pneumonia^18^. In the present study, one of the patients died, although he received anti-*Legionella* drugs. Co-infection with Mimivirus may be influential, as the other three patients, despite an underlying disease, recovered after a period.

Interestingly, our patients had underlying diseases, such as CF, HIV with a low CD4 count, diabetes, and renal disease. However, there is no evidence indicating the relationship between Mimivirus pulmonary infections and underlying diseases. The results of this study showed that individuals with an underlying disease were more likely to be infected with a pulmonary disease due to Mimivirus. However, Mimiviruses are potential pneumonia agents in both non-immunocompromised and immunocompromised patients^19^.

There are three lineages of Mimivirus (A, B, and C), according to the phylogenomic information (using conserved core genes)^1^. Two previous studies have reported that the lineage C of Mimivirus could cause a pulmonary disease^8,20,21^. In agreement with these results, all isolated Mimiviruses in this study were also from lineage C. It seems that lineage C of Mimivirus might be responsible for pulmonary infections.

In line with a study by La Scola^5^, besides real-time PCR assays, the suicide nested-PCR method was used in our study to avoid sample contamination while detecting this rare pathogen. Few studies have shown the presence of Mimivirus DNA in respiratory samples^5,8,21^. In the current study, the virus was detected in both lower and upper respiratory tracts by molecular techniques. Conversely, in several studies, PCR assays could not confirm that Mimivirus could be a human pathogenic agent.

In this regard, Dare et al.^7^ evaluated 496 respiratory specimens for Mimivirus by real-time PCR method, but no positive results were obtained for Mimivirus DNA. Another study evaluated Mimivirus DNA in 214 nasopharyngeal aspirate samples from children with bronchiolitis. This virus was not found in any of the respiratory samples^22^. One of the reasons for the discrepancy between the results could be the small sample size of these studies, compared to our study, besides the significant presence of nucleotide polymorphisms of giant virus genes; this hypothesis was confirmed by the current identification of many Mimiviruses^23^.

The limitation of this study was the lack of *Acanthamoeba polyphaga* strains for preparing antigens for a microimmunofluorescence study.

In conclusion, the results of the current study indicated that the prevalence of Mimivirus was 0.04% in TB-suspected patients. Our findings revealed that Mimivirus was correlated with pneumonia and that it was a potential agent of pneumonia in immunocompromised patients. However, further studies are necessary to confirm the role of Mimivirus pathogenicity in humans.

## Supplementary Information

Supplementary Figures.

Supplementary Tables.

## Data Availability

All data that support all the experimental findings in this article is available in the Supplementary Data File provided. The Mimivirus partial genomes were submitted to GenBank under accession numbers, MN503292, MN503292, MN503294, and MN503295 for Mimivirus-53, Mimivirus-186, Mimivirus-1291, and Mimivirus-1922, respectively.
