# Supplementary material for: Detection of Mimivirus from respiratory samples in tuberculosis-suspected patients
Source: Sci Rep. 2022 May 23;12:8676. doi: 10.1038/s41598-022-12757-6 (PMC9126102; doi:10.1038/s41598-022-12757-6)
Supplement: Supplementary file 1 — Supplementary Figures. [file 41598_2022_12757_MOESM1_ESM.docx]

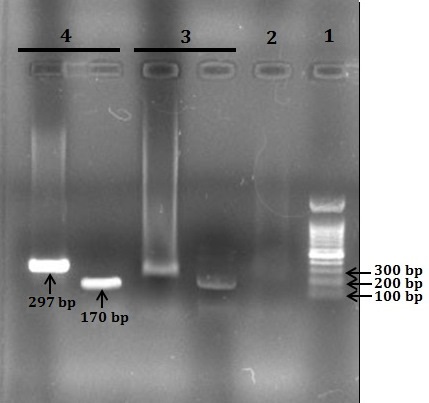


**Supplementary Figure 1:** The first- and second-round PCR products were 297 bp and 170 bp, respectively, visualized by electrophoresis on 1.5% agarose gel. **Line 1:** ladder (100 bp), **Line 2:** Negative control, **Line 3 and 4:** Positive patients.


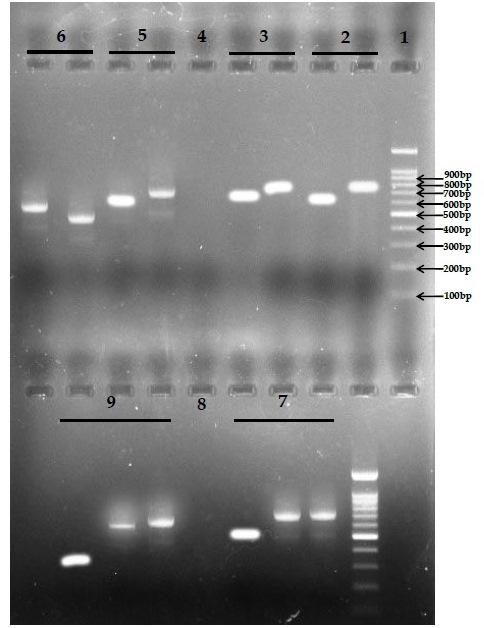


**Supplementary Figure 2A:** The PCR products were VV A18 helicase, major capsid protein, and D5 helicase genes, visualized by electrophoresis on 1.5% agarose gel. **Line 1:** ladder (100 bp), **Line 2:** FO1/RO1: 788 bp, **Line 3:** FO2/RO2: 650 bp (for the VV A18 helicase), **Line 4:** Negative control, **Line 5:** FI1/RI1: 722 bp, **Line 6:** FI2/RI2: 663 bp (for the major capsid protein gene), **Line 7:** FO1/RO1: 728 bp, FO2/RO2: 712 bp, FO3/RO3: 494 bp, **Line 8:** Negative control, **Line 9:** FI1/RI1: 608 bp, FI2/RI2: 583 bp, FI3/RI3: 279 bp (for the D5 helicase)


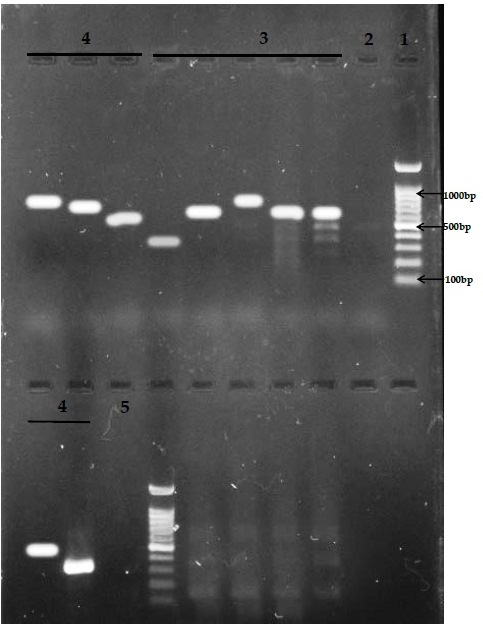


**Supplementary Figure 2B:** The PCR products were the family B-DNA polymerase gene, visualized by electrophoresis on 1.5% agarose gel. **Line 1:** ladder (100 bp), **Line 2:** Negative control, **Line 3:** FO1/RO1: 640 bp, FO2/RO2: 630 bp, FO3/RO3: 809 bp, FO4/RO4: 650 bp, FO5/RO5: 313 bp, **Line 4:** FI1/RI1: 556 bp, FI2/RI2: 624 bp, FI3/RI3: 679 bp, FI4/RI4: 442 bp, FI5/RI5: 271 bp, **Line 5:** Negative control.


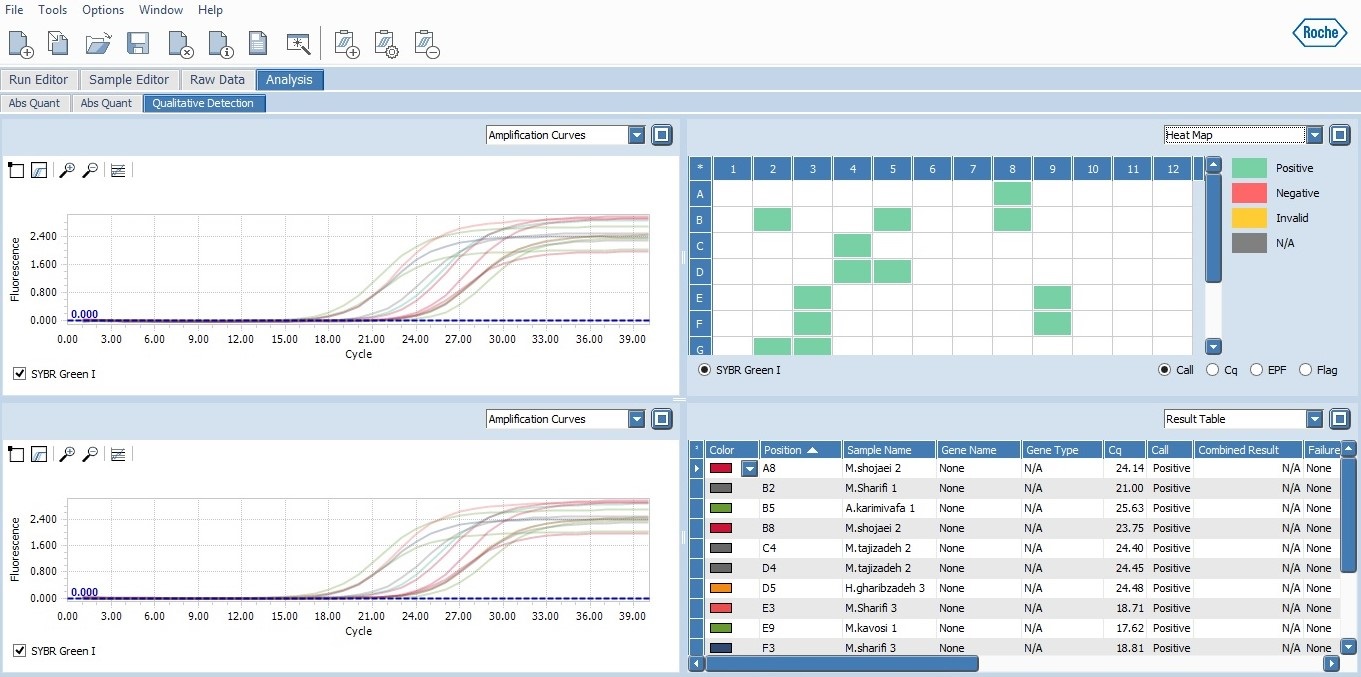


**Supplementary Figure 3:** The raw data for Mimivirus positive samples
