# Supplementary material for: Detection of Mimivirus from respiratory samples in tuberculosis-suspected patients
Source: Sci Rep. 2022 May 23;12:8676. doi: 10.1038/s41598-022-12757-6 (PMC9126102; doi:10.1038/s41598-022-12757-6)
Supplement: Supplementary file 2 — Supplementary Tables. [file 41598_2022_12757_MOESM2_ESM.docx]

**Table S1:** The primer pairs and PCR conditions for amplification of the VV A18 helicase partial-length gene of Megavirus LBA111 (JX885207.1) isolate (1554 bp)

| Fragment |  | Primer position | | | | | | |
| --- | --- | --- | --- | --- | --- | --- | --- | --- |
|  |  | First round PCR | | |  | Second round PCR | | |
|  |  | OuterF |  | OuterR |  | InnerF |  | InnerR |
| 1 |  | 1-18 |  | 767-786 |  | 67-87 |  | 767-786 |
| 2 |  | 553-571 |  | 1183-1202 |  | 892-910 |  | 1536-1554 |

The reaction conditions for the first round of PCR are 35 cycles of 95°C for 40s, 55°C for 30s and 72°C for 1min with a final extension at 72°C for 7min. The same conditions are used for the second round of amplification.

**Table S2:** Primer sequences for the VV A18 helicase gene

| First round PCR (Outer Primers) | Second round PCR (Inner Primers) |
| --- | --- |
| FO1: 5-ATGGTAACAAATGACA-3 | FI1: 5-TATATTGATGATTATGAACTC-3 |
| RO1: 5-TCCATATGTAATATCGGTCC-3 | RI1: 5-TCCATATGTAATATCGGTCC-3 |
| FO2: 5-CCATTTGTAGTAGGTATGG-3 | FI2: 5- CGTTCAGCTATGGTAACTA-3 |
| RO2: 5-GGTGTACCTAAAATTACGAC-3 | RI2: 5-TTATTCACTATCACTTTCC-3 |

**Table S3:** The primer pairs and PCR conditions for amplification of near the family B-DNA polymerase partial-length gene of Megavirus LBA111 (JX885207.1) isolate (4233bp)

| Fragment |  | Primer position | | | | | | |
| --- | --- | --- | --- | --- | --- | --- | --- | --- |
|  |  | First round PCR | | |  | Second round PCR | | |
|  |  | OuterF |  | OuterR |  | InnerF |  | InnerR |
| 1 |  | 1-19 |  | 621-640 |  | 85-101 |  | 621-640 |
| 2 |  | 521-538 |  | 1132-1150 |  | 1068-1085 |  | 1674-1691 |
| 3 |  | 1536-1553 |  | 2323-2344 |  | 2279-2601 |  | 2938-2957 |
| 4 |  | 2834-2853 |  | 3466-3483 |  | 3362-3377 |  | 3784-3802 |
| 5 |  | 3711-3730 |  | 4006-4023 |  | 3963-3982 |  | 4216-4233 |

The reaction conditions for the first round of PCR are 35 cycles of 95°C for 45s, 58°C for 30s and 72°C for 1min with a final extension at 72°C for 7min. The same conditions are used for the second round of amplification.

**Table S4:** Primer sequences for the family B-DNA polymerase gene

| First round PCR (Outer Primers) | Second round PCR (Inner Primers) |
| --- | --- |
| FO1: 5-ATGTTAGTATTTAAAAGTC-3 | FI1: 5-GAACCAATGTTGTATCA-3 |
| RO1: 5-CACTAATACCTTGTCTATCG-3 | RI1: 5-CACTAATACCTTGTCTATCG-3 |
| FO2: 5-TATCCGAGATTAGACCAG-3 | FI2: 5-GGTAGATGATGGATATTC-3 |
| RO2: 5-CTGTTTCTGTAATTCCATC-3 | RI2: 5-TCCCTCATAGAATTTGGA-3 |
| FO3: 5-CTTGATTCCAGTATTAAG-3 | FI3: 5-CTAAAGAAGAGCGTGAAG-3 |
| RO3: 5-CATCATTCATACTTTTGGTAGC-3 | RI3: 5-GAATGATCTTCTGTGACATC-3 |
| FO4: 5-ACGGAGAAGTGTGGACAAGT-3 | FI4: 5-CCTCATACAAATTGTG-3 |
| RO4: 5-TCCAGACTCAGTTTCAAG-3 | RI4: 5-TAGGTGCATTATCACGTCG-3 |
| FO5: 5-GAAATATGTTGGACTTTTG-3 | FI5: 5-GAAACCATCCACAATTGCTC-3 |
| RO5: 5-TCCAGGATCACGAATTGC-3 | RI5: 5-TCTAGAATCAATCATTAG-3 |

**Table S5:** The primer pairs and PCR conditions for amplification of near the D5 helicase partial-length gene of Megavirus LBA111 (JX885207.1) isolate (2757 bp)

| Fragment |  | Primer position | | | | | | |
| --- | --- | --- | --- | --- | --- | --- | --- | --- |
|  |  | First round PCR | | |  | Second round PCR | | |
|  |  | OuterF |  | OuterR |  | InnerF |  | InnerR |
| 1 |  | 19-36 |  | 729-746 |  | 576-594 |  | 1165-1183 |
| 2 |  | 1073-1091 |  | 1767-1784 |  | 1666-1683 |  | 2229-2248 |
| 3 |  | 2127-2146 |  | 2602-2620 |  | 2479-2499 |  | 2738-2757 |

The reaction conditions for the first round of PCR are 35 cycles of 98°C for 20s, 55°C for 30s and 72°C for 1min/kb with a final extension at 72°C for 5min. The same conditions are used for the second round of amplification.

**Table S6:** Primer sequences for the D5 helicase gene

| First round PCR (Outer Primers) | Second round PCR (Inner Primers) |
| --- | --- |
| FO1: 5-CACAAAAACAAAATAGAC-3 | FI1: 5-CCACATACTAACAATATCG-3 |
| RO1: 5-ATCATCAGATCCAGGCAG-3 | RI1: 5-GCCATAGTATAATTACTCG-3 |
| FO2: 5-TCGTTTGTTGACAGATTGG-3 | FI2: 5-ATGGTGTGTATGATTTGG-3 |
| RO2: 5-TTCCATAATGATGCCATC-3 | RI2: 5-CTTCTCCAAGTACCATCATC-3 |
| FO3: 5-GATACTATTACTGCACGTGC-3 | FI3: 5-GTAAGAAATGTGATGTGTTCC-3 |
| RO3: 5-CCAGGACATTTACCATCAT-3 | RI3: 5-CTTTGATATCGTCTAAATTG-3 |

**Table S7:** The primer pairs and PCR conditions for amplification of near the major capsid protein partial-length gene Megavirus LBA111 (JX885207.1) isolate (1625 bp)

| Fragment |  | Primer position | | | | | | |
| --- | --- | --- | --- | --- | --- | --- | --- | --- |
|  |  | First round PCR | | |  | Second round PCR | | |
|  |  | OuterF |  | OuterR |  | InnerF |  | InnerR |
| 1 |  | 34-52 |  | 660-678 |  | 522-541 |  | 894-913 |
| 2 |  | 762-780 |  | 1305-1326 |  | 1208-1227 |  | 1607-1625 |

The reaction conditions for the first round of PCR are 35 cycles of 98°C for 20s, 55°C for 30s and 72°C for 1min/kb with a final extension at 72°C for 5min. The same conditions are used for the second round of amplification.

**Table S8:** Primer sequences for the major capsid protein gene

| First round PCR (Outer Primers) | Second round PCR (Inner Primers) |
| --- | --- |
| FO1: 5-TCGGAAGGAAATCAACTGC-3 | FI1: 5-GATGCTTTTAAATCTGGTGC-3 |
| RO1: 5-CGATTGATTCTTCACCAGT-3 | RI1: 5-CCAATGTAACTGTTTGAATC-3 |
| FO2: 5-CAAGGTGGTAAATTCATGG-3 | FI2: 5-GTTCGATGAAGACAACCGTG-3 |
| RO2: 5-GGAGTTCTACATCAGATACAGG-3 | RI2: 5- CATTCCACTCATGATTCTG-3 |
